# Granulocytic immune infiltrates are essential for the efficient formation of breast cancer liver metastases

**DOI:** 10.1186/s13058-015-0558-3

**Published:** 2015-03-27

**Authors:** Sébastien Tabariès, Véronique Ouellet, Brian E Hsu, Matthew G Annis, April AN Rose, Liliane Meunier, Euridice Carmona, Christine E Tam, Anne-Marie Mes-Masson, Peter M Siegel

**Affiliations:** Goodman Cancer Research Centre, McGill University, 1160 Pine Avenue West, Room 513, Montréal, QC H3A 1A3 Canada; Department of Medicine, McGill University, 3605 Rue de la Montagne, Montréal, QC H3G 2M1 Canada; Department of Biochemistry, McGill University, 3655 Promenade Sir William Osler, Montréal, QC H3G 1Y6 Canada; Centre de Recherche du Centre Hospitalier de l’Université de Montréal (CR-CHUM)/Institut du cancer de Montréal, 900 Saint Denis, Montréal, QC H2X 0A9 Canada; Department of Medecine, Université de Montréal, 2900 Boulevard Edouard-Montpetit, Montréal, QC H3T 1J4 Canada

## Abstract

**Introduction:**

Breast cancer cells display preferences for specific metastatic sites including the bone, lung and liver. Metastasis is a complex process that relies, in part, on interactions between disseminated cancer cells and resident/infiltrating stromal cells that constitute the metastatic microenvironment. Distinct immune infiltrates can either impair the metastatic process or conversely, assist in the seeding, colonization and growth of disseminated cancer cells.

**Methods:**

Using *in vivo* selection approaches, we previously isolated 4T1-derived breast cancer cells that preferentially metastasize to these organs and tissues. In this study, we examined whether the propensity of breast cancer cells to metastasize to the lung, liver or bone is associated with and dependent on distinct patterns of immune cell infiltration. Immunohistocytochemistry and immunohistofluorescence approaches were used to quantify innate immune cell infiltrates within distinct metastases and depletion of Gr1^+^ (Ly-6C and Ly-6G) or specifically Ly-6G^+^ cells was performed to functionally interrogate the role of Ly-6G^+^ infiltrates in promoting metastasis to these organs.

**Results:**

We show that T lymphocytes (CD3^+^), myeloid-derived (Gr-1^+^) cells and neutrophils (Ly-6G^+^ or NE^+^) exhibit the most pronounced recruitment in lung and liver metastases, with markedly less recruitment within bone metastatic lesions. Interestingly, these infiltrating cell populations display different patterns of localization within soft tissue metastases. T lymphocytes and granulocytic immune infiltrates are localized around the periphery of liver metastases whereas they were dispersed throughout the lung metastases. Furthermore, Gr-1^+^ cell-depletion studies demonstrate that infiltrating myeloid-derived cells are essential for the formation of breast cancer liver metastases but dispensable for metastasis to the lung and bone. A specific role for the granulocytic component of the innate immune infiltrate was revealed through Ly-6G^+^ cell-depletion experiments, which resulted in significantly impaired formation of liver metastases. Finally, we demonstrate that the CD11b^+^/Ly-6G^+^ neutrophils that infiltrate and surround the liver metastases are polarized toward an N2 phenotype, which have previously been shown to enhance tumor growth and metastasis.

**Conclusions:**

Our results demonstrate that the liver-metastatic potential of breast cancer cells is heavily reliant on interactions with infiltrating Ly-6G^+^ cells within the liver microenvironment.

**Electronic supplementary material:**

The online version of this article (doi:10.1186/s13058-015-0558-3) contains supplementary material, which is available to authorized users.

## Introduction

Communication between the tumor and surrounding stromal cells is a critical determinant governing the ability of cancer cells to metastasize to specific organs. The tumor microenvironment consists not only of extracellular matrix proteins, resident fibroblasts and endothelial cells, but also infiltrating innate (macrophages, neutrophils, myeloid-derived suppressor cells or natural killer cells) and adaptive (B and T lymphocytes) immune cells [1]. Leukocyte infiltrates are present in the majority of solid tumors; however, the functional roles and clinical consequences of these immune cell infiltrates are complex [2]. In some circumstances, the ability of inflammatory cells to destroy tumor cells has been associated with better prognosis [3,4]. In contrast, numerous studies have shown that inflammation can also contribute to the establishment of primary tumors and subsequent metastasis by allowing tumor cells to escape and/or actively suppress anti-tumor immune responses [2,5,6]. While cancer-related inflammation has mostly been studied in the context of primary tumor growth, it is now accepted that inflammatory cells and secreted mediators are also involved in the migration, invasion and metastasis of malignant cells [2].

Tumor-associated macrophages (TAMs) and myeloid-derived suppressor cells (MDSCs) are well-characterized infiltrating innate cell populations that augment breast cancer metastasis [7], in part, through their ability to stimulate tumor angiogenesis and suppress anti-tumor immunity [8,9]. Infiltration of MDSCs, which are defined as CD11b/Gr-1 double-positive myeloid cells, into the primary tumor and metastatic sites is often associated with poor prognosis in breast cancer patients [10,11]. MDSCs suppress both innate and adaptive immune responses resulting in diminished effector T cell functions [6,12]. In addition, MDSCs also promote the activation and expansion of regulatory T cells to mediate immunosuppression [13]. MDSC accumulation at distant metastatic sites, which contributes to the establishment of a pre-metastatic niche, has also been reported to enhance metastatic efficiency [14]. Thus, the pre-metastatic niche may provide ‘privileged sites’ for tumor cells to adhere and successfully colonize different organs and tissues [15,16].

Neutrophils are garnering attention as important modulators of cancer progression [17-20]. Like TAMs, tumor-associated neutrophils (TANs) may exist in different states of activation/differentiation [21]. TANs can adopt either an anti-tumorigenic (N1) or a pro-tumorigenic (N2) phenotype. Thus, N1-polarized neutrophils have the potential to kill cancer cells and inhibit tumor growth [22-24] as well as coordinate adaptive immune responses through interactions with dendritic cells [25]. In contrast, N2 neutrophils may support tumor growth by producing pro-angiogenic factors and matrix-degrading enzymes, suppress anti-tumor immune responses and facilitate metastasis [21,26-28]. Thus, infiltrating innate immune cell populations, such as TAMs, MDSCs or TANs, can play complex roles during cancer initiation and progression to metastasis. To understand how these cell populations influence general and/or organ-specific breast cancer metastasis, we employed 4T1 breast cancer cell populations isolated from primary mammary tumors, as well as those explanted from bone, lung or liver metastases. Our data reveal that breast cancer cells derived from different metastatic sites are characterized by unique and overlapping patterns of chemokine expression and secretion. Interestingly, liver-metastatic breast cancer cells display high levels of chemokines that recruit a variety of innate immune cell populations. Using antibody-mediated depletion of Ly-6G^+^ cells in immune-competent mice, we uncover a pro-metastatic function for this specific component of the innate immune infiltrate, which is required for the establishment of liver metastases but dispensable for bone and lung metastases.

## Methods

### Cell culture and transfections

The 4T1 cell line was obtained from the American Type Culture Collection and cultured as previously described [29]. The isolation of 4T1-derived cells explanted from primary tumors (148, 152 or 154), 4T1-derived liver-aggressive cell populations (2776, 2792 and 2869), 4T1-derived lung-aggressive cell populations (526, 533 and 537) and 4T1-derived bone-aggressive cell populations (590, 592 and 593) were described previously [29-31].

### RNA amplification, labeling and hybridization to Agilent microarrays

RNA was extracted from 4T1 parental and individual *in vivo* selected metastatic subpopulations, purified total RNA was subjected to amplification and hybridized to 44 K whole mouse genome microarray gene expression chips (Agilent Technologies, Santa Clara, CA, USA) as described previously [30].

### Gene expression analysis

Microarray expression data were normalized and analyzed using GeneSpring software (V7.3, Agilent Technologies). Each group (parental cell line and the explants derived from mammary fat pad, bone, lung and liver metastasis) was individually compared to all the other groups. A two-fold change cutoff was determined and differentially expressed genes were selected using a nonparametric test coupled with a false discovery rate of 0.05. The microarray data can be accessed through the GEO repository (ID GSE62598).

### Bioinformatics analysis

Analysis of differentially expressed genes was performed in the context of gene ontology, canonical pathways and molecular networks using the Ingenuity Pathways Analysis (IPA, Ingenuity Systems™, Qiagen, Redwood City, CA, USA). The differentially expressed genes were compared to genetic categories in the IPA database, and ranked according to *P* values.

### Chemokine array

To detect and quantify chemokines secreted by each cell population, serum-free conditioned media (CM) was collected from cells cultured for 48 h and applied to a chemokine (mouse) quantitative antibody array (Abnova Corp., Taipai, Taiwan, cat#AA0118) following the manufacturer’s protocol. Array scans were acquired with a GenePix 4000B scanner (Molecular Devices, Sunnyvale, CA, USA) at a 5 uM/pixel resolution and features were extracted with the GenePix 6.1 software. The experiment was performed in duplicate.

### Immunohistocytochemistry

For CD3 staining, tissue sections were stained with the Benchmark XT autostainer (Ventana Medical Systems, Inc., Tucson, AZ, USA). Antigen retrieval was performed using Cell Conditioning 1 (Ventana Medical Systems, Inc., cat#950-124) for 60 minutes, following the manufacturer’s instructions. Pre-diluted antibody (CD3: 1:100, Abcam, Cambridge, MA, USA, cat#ab16669) was manually added to the slides and incubated at 37°C for 60 minutes. Reactions were performed using the iView DAB detection kit without secondary antibody (Ventana Medical Systems, Inc., cat#760-093), where pre-diluted anti-rabbit secondary antibody (1:300; Santa Cruz Biotechnology, Dallas, TX, USA) was automatically dispensed. Counterstaining was achieved with hematoxylin and bluing reagent (Ventana Medical Systems, Inc., cat#760-2021 and cat#760-2037).

For Ly-6G or neutrophil elastase (NE) staining, unstained sections (4 μm) were subjected to antigen retrieval in 10 mM citrate buffer (pH 6.0) for 20 minutes at sub-boiling temperatures. Slides were incubated overnight at 4°C with a rabbit anti-Ly-6G antibody (clone 1A8; 1:200 dilution, BD Pharmingen, San Jose, CA, USA, cat#551459) or with a rabbit anti-neutrophil elastase antibody (1:200 dilution, Abcam, cat#ab68672). Following incubation with the primary antibody, secondary biotin-conjugated antibodies were applied for 45 minutes. After washing with phosphate-buffered saline (PBS), slides were developed with diaminobenzidine (Dako, Glostrup, Denmark) as the chromogen and counterstained with Harris hematoxylin. All slides were scanned using a Scanscope XT digital slide scanner (Aperio, Vista, CA, USA). Scans were quantified by analyzing positive staining with Imagescope software (Aperio) using the positive pixel count algorithm. In all analyses, only moderate (+2) and strong (+3) staining was considered as positive pixels. For all staining, data are expressed as a ratio of positive pixels over the total pixels per field.

### Immunofluorescence

Unstained sections (4 μm) were blocked in 1% bovine serum albumin (BSA) with 2% horse serum and 0.05% Tween-20 for 30 minutes. Slides were incubated overnight at 4°C with a goat anti-S100a8 antibody (1:50 dilution, Santa Cruz Biotechnology, cat#sc-8112) and with a rabbit anti-NE antibody (1:50 dilution, Abcam, cat#ab68672). Following incubation with the primary antibody, sections were incubated with Alexa Fluor 555-conjugated donkey anti-goat (cat#A-21432; Molecular Probes, Eugene, OR, USA) and Alexa Fluor 647-conjugated donkey anti-rabbit (Molecular Probes, cat#A-31573) secondary antibodies. Nuclei were counterstained with 4′, 6′-diamidino-2-phenylindole (DAPI), and images were taken (63X) using a Zeiss LSM 710 confocal on an Axio Observer full-motorized inverted microscope (Carl Zeiss Microscopy, Jena, Germany). Images were analyzed with MetaXpress software (Molecular Devices, Sunnyvale, CA, USA). Data is expressed as a percentage of cells double positive for S100a8 and NE over the total number of S100a8-positive cells. Two independent reviewers scored the co-localization of these markers (BEH, CET).

Finally, OCT-embedded livers were sectioned (10 μm) and fixed in methanol for 10 minutes. The sections were blocked with 2% BSA and 1% normal horse serum (Vector Laboratories, Burlingame, CA, USA, cat#S-2000) and subsequently incubated overnight at 4°C with a primary antibody directed against Ly-6G (clone 1A8; 1:100 dilution, BD Pharmingen, cat#561236), CD11b (clone M1/70, 1:100 dilution, eBioscience, San Diego, CA, USA cat#53-0112) and MMP9 (1:100 dilution, Abcam, cat#ab38898). Following incubation with the indicated primary antibodies, Alexa Fluor 555-conjugated donkey anti-rabbit (Molecular Probes, cat#A-31572) secondary antibodies were applied to each section. Nuclei were counterstained with DAPI, and images were taken (63X) using a Zeiss LSM 710 confocal on an Axio Observer full-motorized inverted microscope (Carl Zeiss Microscopy). Images were analyzed with MetaXpress software (Molecular Devices) and the data expressed as a percentage of Ly-6G^+^CD11b^+^MMP9^+^ triple-positive cells (N2-polarized neutrophils) over the total number of Ly-6G^+^CD11b^+^ double-positive cells (neutrophils). Three independent reviewers scored the co-localization of these markers (VO, BEH and ST).

### Experimental metastasis assays

Experimental liver metastasis assays (1 × 10^5^ cells; splenic injection) and experimental lung metastasis assays (5 × 10^5^ cells; tail vein injection) were performed as previously described [30,32]. Tumor burden in the liver (left cardiac lobe) and lungs was quantified as previously reported [30]. The tumor area/tissue area was quantified using Imagescope software (Aperio). Experimental bone metastasis assays (1 × 10^5^ cells; left cardiac ventricle injection) as well as X-ray micro-computed tomography (μCT) imaging was conducted as previously described [33]. All mice were sacrificed two weeks following injection. The mice were housed in facilities managed by the McGill University Animal Resources Centre and all animal experiments were conducted under a McGill University-approved Animal Use Protocol (AUP #5129), which was reviewed by the Facility Animal Care Committee for the Faculty of Medicine (Committee A). All projects involving live animals were subjected to peer review for scientific merit, in accordance with guidelines established by the Canadian Council on Animal Care.

### Antibody-mediated depletion experiments

To deplete Gr-1^+^ cells *in vivo*, purified anti-Ly-6G/Ly-6C rat monoclonal antibody (mAb) (clone RB6-8C5, AbLab, University of British Columbia, BC, Canada) or control isotype immunoglobulin G (IgG) were administered to cohorts of mice. In all cases, mice were treated by intraperitoneal (i.p.) injection (1.5 μg/g) of purified antibodies. Mice were first treated 24 h prior to tumor cell injection and every 72 h thereafter. Differential leukocyte counts were performed at the completion of each experiment and revealed greater than 90% depletion of peripheral blood neutrophils using these treatment regimes.

To specifically deplete Ly-6G^+^ cells *in vivo*, purified anti-Ly-6G rat mAb (clone 1A8, BioXcell, West Lebanon, NH, USA) or control isotype IgG (clone 2A3, BioXcell) were administered to cohorts of mice. Mice were treated by i.p. injection (5.5 μg/g) of purified antibodies as indicated. Briefly, mice were first treated 24 h prior to tumor cell injection and every 48 h thereafter. Differential leukocyte counts were performed at the completion of the experiment and revealed greater than 94% depletion of peripheral blood neutrophils using these treatment regimes. At the experimental endpoint, immunohistochemical staining for Ly-6G revealed up to 65% depletion of liver-infiltrating neutrophils in mice treated with the 1A8 antibody versus the 2A3 isotype control. Quantification of Ly-6G staining was performed as described above (Immunohistocytochemistry).

### CD11b^+^/Ly-6G^+^ cell isolation

Seven days or 14 days following splenic injection of the breast cancer cells, CD11b^+^/Ly-6G^+^ cells were isolated from the metastasis-bearing livers. Livers were first perfused with perfusion medium (Gibco, Waltham, MA, USA, cat#17701-038) for 10 minutes at 5 ml/min. Following perfusion, livers were minced and dissociated for 1 h in a mix of collagenase A, collagenase B and hyaluronidase (2 mg/ml each, Roche, Basel, Switzerland, cat#11088793001 and 11088031001; Sigma-Aldrich, St Louis, MO, USA, cat#H3884).

To purify CD11b^+^/Ly-6G^+^ cells [34,35], cells were blocked in 2.4G2 (1:10 dilution) for 30 minutes and then incubated for 30 minutes, in the dark, with an antibody cocktail containing anti-mouse CD11b-APC (1:10000 dilution, eBioscience, clone M1/70, cat#17-0112-82) and rat anti-mouse Ly-6G-PerCP-Cy5.5 (1:1000 dilution, BD Biosciences, San Jose, CA, USA, clone 1A8, cat#560602). Subsequently, cells were washed once with PBS and then incubated with Live/Dead stain (1:1000 dilution) for 30 minutes. Cells were then washed once with PBS and resuspended in fluorescence-activated cell sorting (FACS) buffer (PBS complemented with 2% fetal bovine serum (FBS)). Samples were read on the BD FACS Canto (BD Biosciences).

### RNA isolation and real-time RT-PCR

Total RNA from isolated CD11b^+^/Ly-6G^+^ neutrophils (isolated as described above) was extracted and reverse transcribed as previously described [30]. Following the reverse transcription reaction, all samples were diluted 1:50 in ddH_2_O and subjected to real-time PCR analysis with FastStart Universal Probe Master (Roche, cat#04913914001). Ten picograms of gene-specific primers (*IFN-*β sense: 5′-TCCATCATGAACAACAGGTG-3′; *IFN-*β antisense: 5′-GACATTTCCGAA TGTTCGTC-3′; *CCL3* sense: 5′-TGTACCATGACACTCTGCAAC-3′; *CCL3* antisense: 5′-CA ACGATGAATTGGCGTGGAA-3′; *TNF-*α sense: 5′-TCGGGGTGATCGGTCCCCAA-3′; *TNF-*α antisense: 5′-GGTGGTTTGCTACGACGTGGGC-3′; *MMP9* sense: 5′-TCGCGTGGAT AAGGAGTTCT-3′; *MMP9* antisense: 5′-CGGTTGAAGCAAAGAAGGAG-3′; *CCL5* sense: 5′-CCTCACCATATGGCTCGGACACC-3′; *CCL5* antisense: 5′- GCGCGAGGGAGAGGTAG GCA-3′; *Arg-1* sense: 5′-CTCCAAGCCAAAGTCCTTAGAG-3′; *Arg-1* antisense: 5′- GGAGCTGTCATTAG GGACATCA-3′) were used in a total reaction volume of 15 μl. For all targets, the following cycling conditions were: 95°C for 10 minutes, followed by 40 cycles each consisting of 95°C for 15 seconds, 60°C for 30 seconds and 72°C for 45 seconds. Incorporation of SYBR Green dye into the PCR products was monitored using a Rotor Gene RG-3000 Real-time PCR system (Roche). Pfaffl analysis method was used to measure the relative quantity of gene expression [36]. The reference gene, *Gapdh* (*Gapdh* sense: 5′-CAAGTATGATGACATCAAGAAGGTGG-3′; *Gapdh* antisense: 5′-GGAAGAGTGGGAGTT GCTGTTG-3′) was selected based on its stable expression in all cell populations analyzed. Relative mRNA levels were expressed as relative expression comparing early CD11b^+^/Ly-6G^+^ neutrophils with late CD11b^+^/Ly-6G^+^ neutrophils. All measurements were done in duplicate from four independent sets of injections.

### Statistical analysis

The significance values associated with the positivity for CD3, Ly-6G (clone 1A8), NE, co-localization of s100a8 and NE and the formation of bone, lung or liver metastases from breast cancer cells in mice depleted for Gr-1^+^ cells or Ly-6G^+^ cells versus isotype controls were calculated using a Student *t* test.

## Results

### Gene expression profiling reveals distinct expression patterns associated with 4T1 subpopulations derived from different metastatic sites

**Figure 1:**
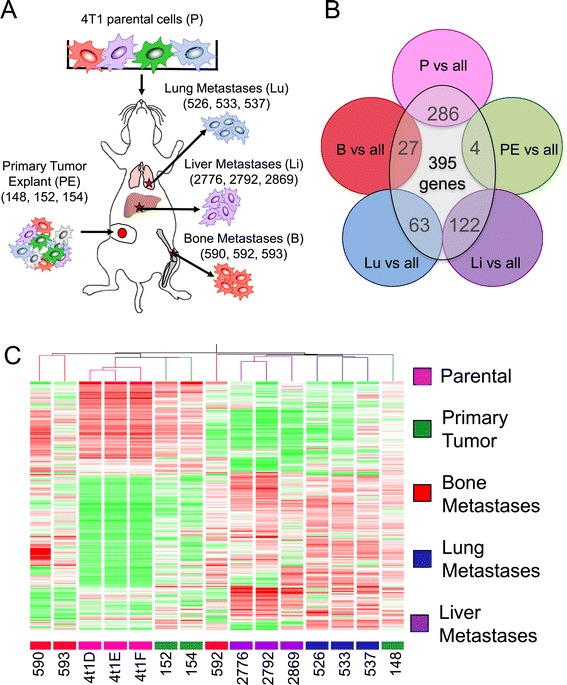
**Gene expression profiling reveals distinct expression patterns associated with different metastatic 4T1 subpopulations. (A)** Schematic depicting 4T1-derived breast cancer cell populations isolated from distinct metastatic sites. **(B)** To identify candidate genes that were differentially expressed among the isolated populations, each group (parental cell line and the explants derived from mammary fat pad (PE), bone, lung and liver metastases) was individually compared to all the other groups. A two-fold change cutoff was determined and 395 differentially expressed genes were selected using a nonparametric test coupled with a 5% false discovery rate. **(C)** A heatmap displaying the hierarchical clustering of isolated 4T1-derived cell populations using the 395 differentially expressed genes. Red color indicates those genes that are highly expressed and the green color denotes those genes that are underexpressed. The majority of cell populations clustered according to the site from which they were derived.

We previously reported the isolation of 4T1-derived subpopulations explanted from primary breast tumors (148, 152 and 154) [31] as well as *in vivo* selected cells that grow aggressively in bone (590, 592 and 593) [29], lung (526, 533 and 537) [31] or liver (2776, 2792 and 2869) [30] (Figure 1A). To identify mediators that contribute to general and/or organ-specific metastatic abilities of breast cancer cells, we performed gene expression profiling using Agilent whole mouse genome microarrays on each of these cell populations (Figure 1B). This approach yielded 395 differentially expressed genes (fold change: 2; false discovery rate: 0.05; see Additional file 1), which, when used to hierarchically cluster the cell populations, could largely segregate them according to the site from which they were derived (Figure 1C).

### 4T1-derived metastatic breast cancer populations possess distinct chemokine profiles

**Figure 2:**
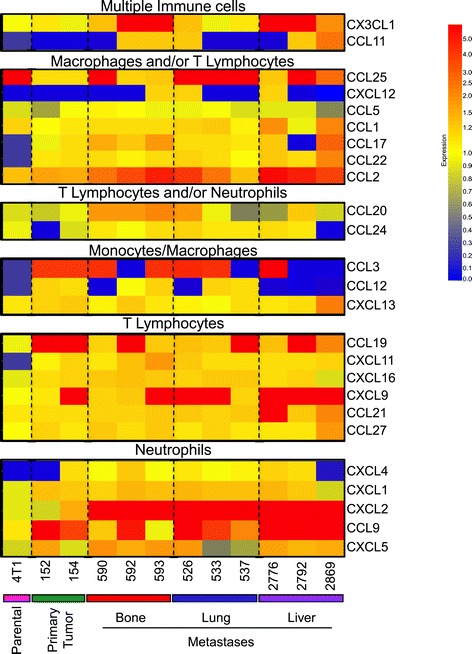
**4T1-derived breast cancer populations possess distinct and overlapping chemokine profiles.** A heatmap representing the secreted levels of 25 chemokines measured using a chemokine array. The chemokines are grouped based on the immune cell types that they are known to recruit (multiple immune cell types, macrophages and/or lymphocytes, T lymphocytes and/or neutrophils, macrophages, T lymphocytes or neutrophils). Red color indicates chemokines that are highly expressed and the blue color denotes chemokines that are underexpressed.

We next subjected the 395 genes to Ingenuity Pathway Analysis (IPA), which revealed 34 significantly affected pathways (Table 1). Of these, a granulocyte adhesion and diapedesis-associated gene signature was the most prominent pathway affected in the various 4T1-derived explants. Intriguingly, other pathways implicating immune cell responses such as IL8 signaling, antigen presentation or leukocyte extravasation were also identified (Table 1). These pathways are characterized by the differential expression of several common genes, including those encoding chemokines (Table 1), which are known to induce directed chemotaxis in responsive leukocyte populations. To determine the repertoire of chemokines secreted by the 4T1-derived breast cancer populations, we employed a multiplex chemokine antibody array (Figure 2). Using this approach, we found that CCL2 and CXCL2, which are chemotactic for inflammatory monocytes and granulocytes [37,38], respectively, were secreted at higher levels by all metastasis-derived cell populations compared to primary tumor explants and the parental cell line (Figure 2). The bone- and lung-aggressive populations exhibited the highest degree of variability in expression of many chemokines; whereas the liver-metastatic populations were also characterized by uniformly high levels of CXCL9 and CCL9 (Figure 2), which are known to recruit T lymphocytes and neutrophils, respectively [39,40]. Collectively, this data demonstrate that cell populations, selected for their ability to metastasize to distant organs, are characterized by an increased production of chemokines and that the pattern of secreted chemokines differs depending on the metastatic site from which they were derived.

**Table 1:** **Differentially affected pathways by Ingenuity Pathway Analysis**

**Ingenuity canonical pathways**	**p-value**	**Ratio**	**Molecules**
***Granulocyte adhesion and diapedesis***	4.94E-06	7.18E-02	VCAM1,SDC1,MMP3,MMP10,CLDN7,CCL5,CXCL5,IL18RAP,CXCL10,ITGB2,MMP9,CLDN3,MMP19
Inhibition of matrix metalloproteases	3.54E-05	1.50E-01	SDC1,MMP3,RECK,MMP10,MMP9,MMP19
MMP19 hepatic fibrosis/hepatic stellate cell activation	8.91E-05	6.45E-02	02VCAM1,CTGF,FGFR1,PDGFRA,LBP,CCL5,PDGFC,MMP9,PGF,IL18RAP
Regulation of the epithelial-mesenchymal transition pathway	1.81E-04	5.61E-02	MAP2K6,CDH1,JAG2,WNT10B,ESRP2,FGFR1,PARD6G,JAG1,NOTCH1,MMP9,CLDN3
***Agranulocyte adhesion and diapedesis***	2.09E-04	5.76E-02	CXCL10,ITGB2,VCAM1,MMP3,MMP10,CLDN7,CCL5,CXCL5,MMP9,CLDN3,MMP19
HIF1alpha signaling	2.68E-04	7.14E-02	MMP3,MMP10,PDGFC,LDHA,MMP9,LDHB,PGF,MMP19
***Antigen presentation pathway***	3.05E-04	1.19E-01	B2M,PSMB9,HLA-B,PSMB8,TAP1
Bladder cancer signaling	5.99E-04	7.22E-02	CDH1,MMP3,MMP10,PDGFC,MMP9,PGF,MMP19
***IL-8 signaling***	9.59E-04	4.44E-02	ITGB2,GNB4,CDH1,ANGPT2,VCAM1,CCND2,MYL12B,PDGFC,MMP9,PGF
***Leukocyte extravasation signaling***	1.48E-03	4.76E-02	ITGB2,VCAM1,EDIL3,MMP3,CD44,MMP10,CLDN7,MMP9,CLDN3,MMP19
VDR/RXR activation	1.63E-03	6.82E-02	CXCL10,SPP1,GADD45A,SEMA3B,VDR,CCL5
Pyruvate fermentation to lactate	2.53E-03	2.22E-01	LDHA,LDHB
ILK signaling	3.47E-03	4.39E-02	MAP2K6,ITGB2,PARVB,CDH1,VIM,PDGFC,MMP9,DSP,PGF
Pathogenesis of multiple sclerosis	8.71E-03	2.00E-01	CXCL10,CCL5
Sphingosine-1-phosphate signaling	8.97E-03	4.88E-02	PLCD1,CASP12,CASP1,PDGFRA,CASP4,PDGFC
Axonal guidance signaling	1.04E-02	2.90E-02	WNT10B,BDNF,MMP10,ADAMTS9,PDGFC,SEMA4C,PGF,PLCD1,GNB4,MYL12B,BMP7,SEMA3B,MMP9,SEMA7A
Atherosclerosis signaling	1.39E-02	4.35E-02	ITGB2,VCAM1,MMP3,S100A8,PDGFC,MMP9
Sorbitol degradation I	1.62E-02	2.00E-01	SORD
Asparagine biosynthesis I	1.62E-02	1.25E-01	ASNS
Alanine biosynthesis III	1.62E-02	3.33E-01	NFS1
Colorectal cancer metastasis signaling	1.73E-02	3.36E-02	GNB4,CDH1,WNT10B,MMP3,MMP10,PDGFC,MMP9,PGF,MMP19
p53 signaling	1.75E-02	4.63E-02	TP53INP1,CCND2,GADD45A,PLAGL1,SFN
Role of IL-17A in psoriasis	1.81E-02	1.43E-01	S100A8,CXCL5
Chondroitin sulfate degradation (metazoa)	2.09E-02	8.70E-02	CD44,HEXA
Human embryonic stem cell pluripotency	2.19E-02	3.73E-02	WNT10B,BDNF,FGFR1,PDGFRA,BMP7,PDGFC
Notch signaling	2.33E-02	6.98E-02	JAG2,JAG1,NOTCH1
Dermatan sulfate degradation (metazoa)	2.38E-02	8.70E-02	CD44,HEXA
Thyroid cancer signaling	2.66E-02	6.82E-02	CXCL10,CDH1,BDNF
Alanine degradation III	3.21E-02	1.67E-01	GPT2
Alanine biosynthesis II	3.21E-02	1.67E-01	GPT2
Formaldehyde oxidation II (glutathione-dependent)	3.21E-02	1.00E-01	ESD
Glutamine degradation I	3.21E-02	2.00E-01	GLS2
GADD45 signaling	4.09E-02	8.33E-02	CCND2,GADD45A

### T lymphocytes are recruited to breast cancer liver and lung metastases

**Figure 3:**
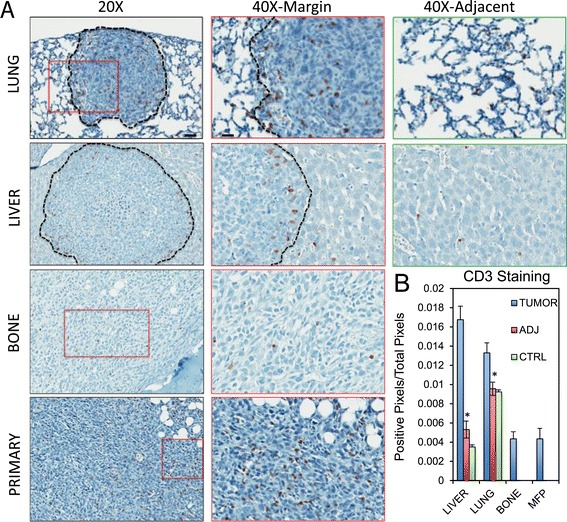
**T lymphocytes are recruited to liver and lung metastases from breast cancer.** Paraffin-embedded sections from primary breast tumors, bone specimens, lung specimens and liver specimens obtained following experimental metastasis assays were subjected to immunohistochemical staining with anti-CD3 antibodies. **(A)** Representative images from 20X, 40X magnifications for each site are shown. 40X images were taken at the margin of the lesions (40X margin) and in more distal regions (40X adj.). Scale bar represents 40 μm (20X) or 20 μm (40X) and applies to all panels of the same magnification. **(B)** Positivity of CD3 staining (expressed as a ratio of positive pixels over the total pixels per field) quantified inside lesions (TUMOR), in the adjacent tissue (ADJ) or in control (CTRL) samples without any lesions. Lymphocyte T expression and recruitment is mostly associated with lung or liver metastasis (^*^: *P* <0.001).

Metastatic breast cancer cells expressed chemokines that can direct the recruitment of T lymphocytes (CXCL9, CCL2 or CX3CL1) [40-42]. Immunohistocytochemistry staining with an antibody against a pan T cell marker (CD3) revealed positivity in all samples examined (Figure 3A,B). Indeed, CD3-positive staining was found enriched in both lung and liver metastases compared to bone metastases or primary tumors (Figure 3B). In addition, CD3-positivity was higher in lung or liver metastases compared to tissue adjacent (ADJ) to the metastases or control (CTRL) samples that were devoid of lesions (Figure 3B). However, while CD3-positive cells were found throughout the lung lesions, about 2/3 of liver lesions revealed increased abundance of CD3-positive cells at the periphery of the metastatic lesions (Figure 3). These data indicate that T lymphocyte recruitment is associated with lung and liver metastases and localization differs depending on metastatic site.

### A granulocytic infiltrate is recruited in breast cancer-derived lung and liver metastases

**Figure 4:**
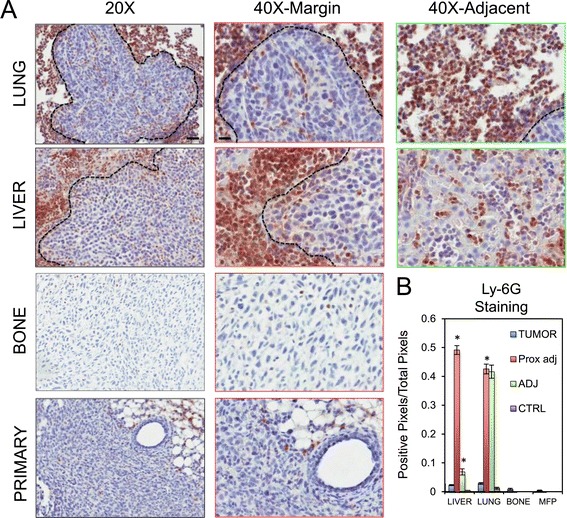
**Ly-6G** ^**+**^ **cells are recruited in breast cancer-derived lung and liver metastases.** Paraffin-embedded sections from primary breast tumors, bone metastases, lung metastases and liver metastases were obtained following experimental metastasis assays and subjected to immunohistochemical staining with anti-Ly-6G antibodies. **(A)** Representative images from 20X and 40X magnifications for each metastatic site are shown. 40X images were taken either at the margin of the lesions (40X margin) or in regions distal to the metastatic lesion (40X adj.). **(B)** Positivity of Ly-6G staining (expressed as a ratio of positive pixels over the total pixels per field) was quantified within the metastatic lesions (TUMOR), in close proximity to the metastatic lesion (PROX ADJ), in the tissue adjacent (ADJ) to the metastases or in control (CTRL) samples without any metastatic lesions. Increased recruitment of Ly-6G+ cells was observed in close proximity to hepatic metastases (^*^: liver tumor vs. liver adj., *P* <0.001; liver adj. vs. liver prox. Adj., *P* <0.001; lung tumor vs. lung adj., *P* <0.001). Scale bar represents 40 μm (20X) or 20 μm (40X) and applies to all panels of the same magnification.

Several chemokines that are known to recruit myeloid cells (CCL2, CCL9, CXCL2), including monocytes and neutrophils, were expressed by metastatic breast cancer cells (Figure 2) [39,43]. We next extended our analysis by performing immunohistocytochemical staining on 4T1-derived metastatic samples with a Ly-6G antibody (clone 1A8). While some data suggests that Ly-6G can be expressed on granulocytic MDSCs [44,45], multiple studies suggest that Ly-6G serves as a neutrophil marker [46-50]. Low to moderate Ly-6G staining was observed in primary tumors and bone metastases. In contrast, strong staining was observed in lung and liver metastases compared to control (CTRL) samples that were devoid of lesions (Figure 4). Interestingly, while some positive cells were found within the metastatic lesions (TUMOR), the vast majority of positive cells were located in tissue adjacent (ADJ) to the metastases (Figure 4). Strikingly, a very high density of Ly-6G-positive cells was observed in close proximity (PROX ADJ) of the lesions, specifically at the margin surrounding hepatic metastases, compared to more distal tissue (ADJ, Figure 4). These data reveal a significant granulocytic component in liver metastases formed by 4T1 liver-metastatic breast cancer cells.

We next confirmed these observations by staining the metastatic samples with an antibody against NE as an additional neutrophil marker (see Additional file 2) [51,52]. In agreement with the Ly-6G staining, low to moderate NE staining was observed in primary tumors and bone metastases, while stronger NE staining was detected in lung and liver metastases compared to CTRL tissues lacking metastatic lesions (see Additional file 2). Moreover, NE positivity was mainly detected in tissue adjacent to the lesions (PROX ADJ) with the most intense staining localized immediately next to the liver metastases (PROX ADJ) (see Additional file 2). Taken together, our results demonstrate differential recruitment of immune cells into distinct metastatic sites, with high granulocyte and neutrophil positivity being detected in bone marrow, lung tissue and liver tissue. However, the pattern of infiltrations is different depending upon the metastatic lesion in question.

### Gr-1^+^ cell depletion differentially affects the formation of bone, lung and liver metastases

**Figure 5:**
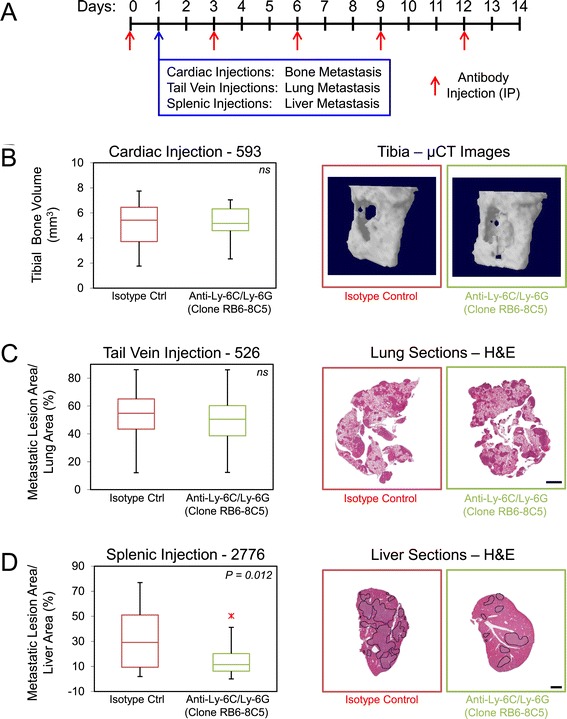
**Depletion of Gr-1** ^**+**^ **(Ly-6C/Ly-6G) cells differentially affects the establishment and growth of bone, lung and liver metastases. (A)** Schematic depicting the experimental protocol for depletion of Gr-1^+^ cells. **(B)** The degree of osteolytic bone destruction in the hindlimbs of mice treated with anti-Gr1 and isotype control antibodies was quantified by *in vivo* micro-computed. tomography (μCT) imaging. Bone volume of defined regions of the proximal tibia is shown following cardiac injection of 593 bone-aggressive cells. No difference was observed when comparing bone volumes between the two populations. Representative images of bone reconstructions are shown. **(C)** Quantification of the tumor burden (tumor area/tissue area) within the lung following tail vein injection of 526 lung-aggressive cells. No statistical difference in lung metastatic burden was observed when the isotype control cohort was compared with Gr-1^+^-depleted cohort. **(D)** Quantification of the tumor burden (tumor area/tissue area) within the cardiac liver lobe following splenic injection of 2776 liver-aggressive cells. Statistically significant decreases in liver-metastatic burden were observed when the isotype control cohort was compared with the Gr-1-depleted cohort (*P* = 0.012). Hematoxylin and eosin (H&E) images of the lung or cardiac liver lobe are shown for mice injected with each of the indicated cell populations and treated with isotype control or anti-Gr1 antibodies. Dotted lines circumscribe breast cancer metastatic lesions within the liver. Scale bar represents 2 mm and applies to all panels. IP: intraperitoneal injection; ns: not statistically significant.

Our data reveal a significant innate immune infiltrate in soft tissue metastases. To examine whether monocytes/macrophages or granulocytic cells present in these infiltrates can influence the metastatic abilities of the 4T1-derived subpopulations, we first assessed the ability of 4T1 metastatic breast cancer cells to establish and grow in mice depleted of monocytes/macrophages, MDSCs, dendritic cells and neutrophils (Figure 5A). Treatment of mice with anti-Ly-6C/Ly-6G antibodies (clone RB6-8C5) is a well-established method that results in the depletion of approximately 97% of circulating neutrophils [20,53,54]. Consistent with the immunohistochemical staining results, depletion of Ly-6C^+^/Ly-6G^+^ cells had no major effect on the overall bone metastatic burden, as measured by μCT analysis, following left cardiac ventricle injection of bone-aggressive breast cancer cells (Figure 5B). We also interrogated whether Ly-6C^+^/Ly-6G^+^ cells support lung and liver breast cancer metastasis given the significant enrichment of these immune cells into (lung) or surrounding (liver) metastatic nodules. Surprisingly, depletion of Ly-6C^+^/Ly-6G^+^ cells also had no effect on the establishment of lung metastasis following intravenous injection of lung-aggressive cells (Figure 5C). In contrast, following splenic injection of the liver-aggressive 4T1 subpopulations, Ly-6C^+^/Ly-6G^+^-depleted mice showed markedly reduced tumor burden in the liver when compared to their control counterparts (Figure 5D). Indeed, lesions formed in control mice occupied 2.36 times less of the liver area when compared to control mice (Figure 5D).

**Figure 6:**
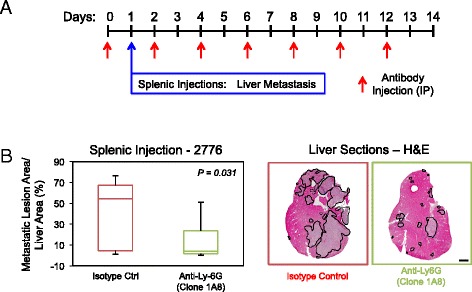
**Ly-6G** ^**+**^ **cell depletion decreases the establishment and growth of liver metastases. (A)** Schematic depicting the experimental protocol for depletion of Ly-6G^+^ cells. **(B)** Paraffin-embedded liver sections were obtained following completion of the experimental metastasis assays and subjected to immunohistochemical staining with anti-Ly-6G antibodies. Statistically significant decreases in Ly-6G^+^ cells that infiltrate the liver were observed when the neutrophil-depleted cohort was compared with the isotype control cohort (*P* = 0.045). Representative images of Ly-6G-stained liver sections from each cohort. **(C)** Quantification of the tumor burden (tumor area/tissue area) within the cardiac liver lobe following splenic injection of 2776 liver-aggressive cells. Statistically significant decreases in liver-metastatic burden were observed when the isotype control cohort was compared with the neutrophil-depleted cohort (*P* = 0.031). Representative images of hematoxylin and eosin (H&E)-stained liver sections exhibiting the liver-metastatic burden in each cohort. Dotted lines circumscribe breast cancer metastatic lesions within the liver. Scale bar represents 2 mm and applies to all panels. IP: intraperitoneal injection.

The use of anti-Ly-6C/Ly-6G antibodies in the previous depletion experiment will remove both monocytes/macrophages and granulocytes [21,49,50,55,56]. To narrow down the critical cell population required for the efficient formation of liver metastases, we next examined the ability of the liver-aggressive 4T1 subpopulations to establish and grow in the liver of mice depleted using anti-Ly-6G antibodies (clone 1A8) (Figure 6A). Indeed, Ly-6G has been shown to be specifically expressed by granulocytes or polymorphonuclear (PMN) MDSCs [44]. The use of anti-Ly-6G antibodies (clone 1A8) has been widely and extensively used in the literature to deplete neutrophils [21,49,55-59]. Immunohistochemical staining using anti-Ly-6G antibodies revealed a 2.7-fold decrease in neutrophils that were recruited to the liver at the experimental endpoint (Figure 6B). As observed with mice depleted of Ly-6C^+^/Ly-6G^+^ cells, depletion of only Ly-6G^+^ cells resulted in decreased tumor burden in the liver following splenic injection, resulting in a 2.92-fold reduction in the liver-metastatic area/liver tissue area when compared to mice treated with the isotype control antibodies (Figure 6C). Together, these data demonstrate that the recruitment of Ly-6G^+^ granulocytes, of which neutrophils comprise a significant component, is functionally involved in breast cancer cell metastasis to specific sites, being dispensable for the formation of bone and lung metastases but playing an important positive role in the colonization and growth of liver metastases.

### Neutrophil recruitment persists at the lesion margins during progression of breast cancer-derived liver metastases

**Figure 7:**
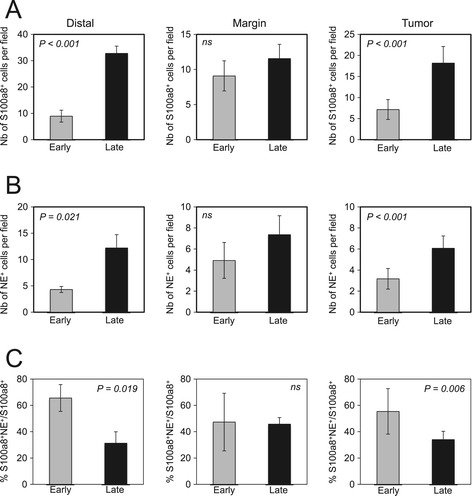
**Neutrophils are recruited to early lesions and are maintained at the margin of liver metastases over time.** Paraffin-embedded sections from liver metastases were collected at early (1.5 week) or late (3 weeks) time points following experimental metastasis assays and subjected to immunohistofluorescence staining with anti-S100a8 (red) or anti-neutrophil elastase (NE) (green) antibodies. The number of S100a8-positive cells **(A)** or NE-positive cells **(B)** per field was quantified. **(C)** Positivity of neutrophil staining (expressed as a percentage of S100a8 and NE double-positive cells over the total number of S100a8-positive cells) was quantified within each region of interest. While the number of s100a8- and NE-positive cells increased during tumor progression (s100a8 distal early vs. distal late, *P* <0.001; NE distal early vs. distal late, *P* = 0.021; s100a8 tumor early vs. tumor late, *P* <0.001; NE tumor early vs. tumor late, *P* <0.001), a decrease in the proportion of neutrophils comprising the s100a8^+^ infiltrate was routinely observed in the tumor or the distal region as tumor progressed (distal early vs. distal late, *P* = 0.019; tumor early vs. tumor late, *P* = 0.006). In contrast, no changes were observed in the margin of the lesions.

Given that the tumor microenvironment evolves as tumors progress [60], we assessed if the observed recruitment of neutrophils changed during hepatic metastasis progression. To do so, we examined liver metastases samples isolated from mice either at early (10 days) or late (21 days) time points post splenic injection with 2776 or 2792 liver-aggressive cell lines. We performed immunohistofluorescence using S100a8 as a marker whose expression is restricted to macrophages, MDSCs and neutrophils [61-64] coupled with NE as a marker for neutrophils [51,52]. We observed a clear increase in the total numbers of s100a8^+^ (Figure 7A) and NE^+^ (Figure 7B) cells within the lesions and in the distal liver, with minimal changes at the margin of the liver metastases (Figure 7A,B). However, the proportion of the s100a8^+^ infiltrate that is composed of neutrophils decreased over time within the metastatic lesions (TUMOR; Figure 7C, see Additional file 3) and in distal tissue (DISTAL; Figure 7C), suggesting the late recruitment of additional innate immune cell populations. Interestingly, no major changes in the percentage of neutrophils were observed at the margin surrounding hepatic metastases (MARGIN, Figure 7C, see Additional file 3). Together, our results suggest that recruitment of NE^+^ neutrophils at the invasion front of liver metastasis is an early event that is maintained during liver metastases progression. The initial infiltration of neutrophils is followed by a subsequent influx of s100a8^+^ cells that may include macrophages, MDSCs and additional granulocytic cells.

### N2-polarized neutrophil recruitment increases at the lesion margins during progression of breast cancer-derived liver metastases

**Figure 8:**
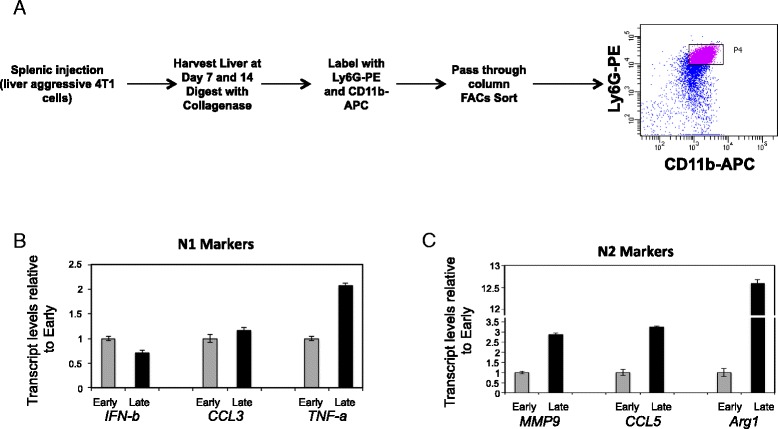
**N2-polarized neutrophils are recruited to the invasive front of liver metastases over time. (A)** Schematic depicting the experimental protocol to isolate neutrophils from metastasis-bearing livers. **(B)** Quantitative real-time PCR analysis was performed for *IFN-*β*, CCL3* and *TNF-*α as markers for anti-tumorigenic (N1)-polarized neutrophils, normalized to total *Gapdh* levels, in neutrophils isolated from early (7 days) or late (14 days) time points post splenic injection with the 2776 liver-aggressive cell line. The data is depicted as fold expression relative to early time point and is representative of four independent experiments performed in triplicate. **(C)** Quantitative real-time PCR analysis was performed for *MMP9, CCL5* and *Arginase 1* as markers for pro-tumorigenic (N2)-polarized neutrophils, normalized to total *Gapdh* levels, in neutrophils isolated from early or late time points post splenic injection with the 2776 liver-aggressive cell line. The data is depicted as fold expression relative to early time point and is representative of four independent experiments performed in triplicate.

In order to characterize the phenotype of neutrophils during liver metastasis development, we isolated these cells based on CD11b and Ly-6G expression, which are routinely used in the literature as neutrophil markers [21,44,48,55,58,65]. Liver-infiltrating neutrophils were purified from mice at early (7 days) or late (14 days) time points following splenic injection with the 2776 liver-aggressive cell line (Figure 8A). To study the phenotypic changes that occurred in the neutrophils during liver metastasis progression, we performed real-time quantitative PCR (qPCR) to monitor the expression of genes previously reported to differentiate N1- and N2-polarized neutrophils [60,66]. With the exception of *TNF-α*, we did not detect significant differences in the expression of molecules reported to be preferentially expressed in N1-polarized neutrophils, such as *IFN-β* or *CCL3*, when comparing early and late isolated liver metastasis-associated neutrophils (Figure 8B). However, we did observe an increase in the expression of molecules reported to be preferentially expressed in N2-polarized neutrophils, such as *MMP9*, *CCL5* or *Arginase 1*, which were increased during liver metastasis progression (2.88-, 3.23- and 12.59-fold, respectively) (Figure 8C). These data suggest that some neutrophils have undergone a putative transition to an N2 phenotype while others retain markers suggestive of an N1 phenotype. Such a result could indicate that neutrophil phenotypes could be influenced by their location within the liver and their proximity to the metastatic lesions.

Since our results highlighted the recruitment of neutrophils at the invasive front of liver metastases during liver metastasis progression (Figure 7), we next assessed if N2-like tumor-educated neutrophils are enriched in the invasion front compared to more distal regions. Thus, we examined liver metastases isolated from mice either at early (7 days) or late (14 days) time points post splenic injection with 2776 liver-aggressive cell line and performed immunofluorescence using CD11b and Ly-6G (clone 1A8) as markers for neutrophils coupled with MMP9 as a marker associated with N2-polarized neutrophils. We observed a clear increase in the percentage of N2-polarized neutrophils (defined as CD11b^+^/Ly-6G^+^/MMP9^+^) at the invasive front of liver metastases compared to those situated in regions distal to the metastatic lesions (Table 2, see Additional files 4 and 5). Moreover, we observed a correlation between the percentage of N2-polarized neutrophils and the liver-metastatic burden (Table 2). Taken together, our results support the presence of a mixed population of neutrophils during breast cancer liver metastasis development and demonstrate that neutrophils become more N2 polarized as liver metastasis progresses and a greater fraction of neutrophils adopt an N2 phenotype when they are located close to the liver-metastatic lesions.

**Table 2:** **N2-polarized neutrophils are recruited at the invasive front of breast liver metastases over time**

		**D7**	
**Location**	**0 < metastatic lesion area/liver area (%) <1.79**		
	**% N2 neutrophils** ^**a**^	**Error**	***P*** **value**
Distal	60.84	+/− 9.39	
Invasive front	90.19	+/− 6.04	<0.00001
		**D14**	
**Location**	**Low metastatic burden**		
	**2.16 < metastatic lesion area/liver area (%) <4.19**		
	**% N2 neutrophils** ^**a**^	**Error**	***P*** **value**
Distal	79.58	+/− 5.93	
Invasive front	94.92	+/− 3.56	<0.0001
		**D14**	
**Location**	**High metastatic burden**		
	**13.62 < metastatic lesion area/liver area (%) <54.12**		
	**% N2 neutrophils** ^**a**^	**Error**	***P*** **value**
Distal	88.21	+/− 3.53	
Invasive front	93.63	+/− 2.80	0.003

^a^OCT-embedded sections from liver metastases were collected either 7 days (D7) or 14 days (D14) following splenic injection of breast cancer cells and subjected to immunohistofluorescence staining with anti-Ly-6G (cyan), MMP9 (red) or Cd11b (green) antibodies. The percentage of pro-tumorigenic (N2)-polarized neutrophils (Cd11b^+^/Ly-6G^+^/MMP9^+^) out of the total number of neutrophils (Cd11b^+^/Ly-6G^+^) was assessed either at the invasive front of the metastatic lesions or in regions distal to the metastases (distal). Representative images can be found in Additional files 3 and 4.

## Discussion

We have employed 4T1 breast cancer subpopulations, derived from primary mammary tumors or bone, lung and liver metastases, to identify potential mediators of general or organ-specific breast cancer metastasis. Ingenuity Pathways Analysis highlighted differential expression of genes involved in several pathways implicating immune responses. This observation is not surprising considering that leukocyte infiltrates are detected in most tumors and are often associated with poor prognosis [2,67]. Distinct chemokines secreted by cancer cells are responsible for recruiting immune cells to the growing tumor, and the composition of this infiltrate can change during cancer progression [68]. Our results reveal that secretion of distinct and overlapping chemokines characterizes breast cancer cell populations isolated from different metastatic sites. Of these, CCL2 and CXCL2 were uniformly elevated in all of the metastatic populations, regardless of origin. CCL2 is expressed in various cancers, including breast tumors, and is considered a tumor-derived chemotactic factor for diverse immune cell types [9]. CCL2 production, by the tumor and/or cells within the stromal microenvironment, recruits Gr-1^+^ cells to breast cancer lung metastases and leads to the subsequent recruitment of metastasis-associated macrophages [38]. Moreover, it has been shown that CCL2 expression by colorectal cancer cells can recruit Gr-1^+^ cells, which are important for the establishment and growth of colorectal liver metastases [69,70]. Conversely, CCL2 has been implicated in the recruitment of neutrophils into lungs of mice bearing breast cancer lung metastases, which eliminate disseminated cancer cells and diminish the formation of metastases [71]. Thus, the ultimate consequence of CCL2-mediated immune cell recruitment, either pro- or anti-metastatic, will be highly context dependent.

Considerably less is known about the functional roles played by CXCL2 in breast cancer metastasis. Recently, it was shown that breast cancer cells expressing elevated levels of CXCL1 and CXCL2 induce the recruitment of MDSCs into the primary mammary tumor. These infiltrating immune cells secrete s100a8/s100a9 that augments breast cancer survival during metastasis and in response to chemotherapy. The use of CXCL1/2 receptor inhibitors, combined with chemotherapy, resulted in reduced lung metastasis using MDA-MB-231 breast cancer cells [72]. These observations indicate that metastatic cancer cells secrete cytokines, such as CCL2 and CXCL2, to recruit innate immune cells that drive metastasis formation.

Interestingly, CCL9 and CX3CL1 were strongly and uniformly elevated in the liver-metastatic breast cancer cells. CCL9 (and CCL15) secreted by mouse and human colon cancer cells has been shown to recruit immature myeloid cells (iMCs), which produce matrix metalloproteinases MMP2 and MMP9 required for successful colonization of the liver [73]. Similarly, using a TGFβ signaling-deficient model of colon cancer, it was shown that increased numbers of iMCs are recruited from the bone marrow to the invasive front of liver metastases, in response to a gradient of CCL9 [74]. It has also been recently shown that CX3CR1, the receptor for CX3CL1, is functionally required in TAMs to support angiogenesis and efficient formation of colon cancer liver metastases [75]. Together, these observations are consistent with the selection for elevated expression of CCL9 and CX3CL1 in breast cancer cells that are metastatic to the liver.

Our immunohistochemical staining revealed that a significant proportion of cells detected in metastatic tissues are neutrophils, as determined by Ly-6G or NE staining. The enrichment of neutrophils at early stages and the recruitment of additional innate immune cells (s100a8^+^) at later time points was further confirmed by our immunofluorescence data. Interestingly, s100a8 and s100a9, which can be released from neutrophils, act as strong chemoattractants for the subsequent recruitment of macrophages and activated monocytes [76,77]. We observed that the proportion of NE^+^ neutrophils within the innate immune infiltrate diminished during the growth of liver metastases (within the lesions and in distal liver tissue), but were retained at the invasive front of the growing metastases. Our data reveal that neutrophils become increasingly polarized toward an N2 phenotype as the liver metastases progress. Furthermore, we demonstrate that neutrophils situated at the margin of the liver-metastatic lesions are enriched for ‘N2’ polarized phenotype compared to those located distal to the metastases. The latter observation is in agreement with previous findings that TANs, in the context of mesothelioma, acquire a more pro-tumorigenic phenotype as the tumors progress [60]. Thus, it is possible that infiltrating neutrophils, and the subsequent recruitment of MDSCs, are needed for the progression of liver metastases by dampening anti-tumor immune responses and promoting a pro-angiogenic microenvironment [78].

We demonstrate that depletion of Gr-1^+^ cells in mice differentially affects the metastatic potential of well-characterized 4T1 breast cancer cell populations [29-31]. Previous reports have shown that depletion experiments, using Gr-1-specific (Ly-6C/Ly-6G) antibodies, inhibit tumor growth, reduce endothelial cell recruitment to tumors and limit metastasis, in part, by restoring immune surveillance [28,79-83]. In contrast, it has been reported, using similar Ly-6G-specific depletion experiments, that removal of neutrophils can promote the formation of lung metastases [71]. We did not observe any positive or negative effects on the formation of lung or bone metastases following Ly-6C/Ly-6G-mediated depletion. In contrast, depletion of either Ly-6C^+/^Ly-6G^+^ cells or specifically Ly-6G^+^ cells reduced the ability of liver-aggressive breast cancer populations to form hepatic metastases, supporting a pro-metastatic role for neutrophils in this process. These data argue that, despite a complex innate immune infiltrate surrounding the emerging liver metastases, it is the neutrophil component (Ly-6G^+^ cells) that plays a critical functional role in the establishment and growth of 4T1-derived liver metastases. An emerging body of literature supports a role for MDSCs and neutrophils in specifically promoting liver metastasis [78]. Colorectal cancer liver metastases are characterized by the infiltration of bone marrow-derived CD11b/Gr-1^mid^ cells, in response to CCL2 secreted by CRC cells [69,70].

Roles specific to neutrophils and liver metastasis are also emerging. Indeed, neutrophils have been implicated in facilitating tumor cell extravasation. In the presence of inflammatory stimuli, neutrophils have been shown to adhere to activated sinusoidal endothelium and serve as a bridge to capture circulating tumor cells to promote seeding within the liver [20,84,85]. In the context of systemic inflammation, administration of a NE inhibitor decreased the liver-metastatic burden following injection of lung carcinoma cells. In this study, neutrophils were stimulated to undergo a process called NETosis, which is the expulsion of condensed chromatin that forms extracellular nets, caused the entrapment of circulating tumor cells and enhanced the formation of liver metastases [19]. These data argue that neutrophils perform metastasis-promoting functions immediately upon infiltration into the metastatic site. This idea is challenged by studies that show neutrophils recruited to the lung can facilitate clearance of disseminated tumor cells, resulting in a reduction in lung metastasis [71]. This concept was reinforced by the studies demonstrating that early recruitment of neutrophils impaired the growth of mesothelioma or Lewis Lung carcinomas via cytotoxic anti-tumor effects (N1 phenotype). Subsequently, tumor-derived factors polarized neutrophils toward a pro-tumorigenic (N2 phenotype) in response to tumor-derived factors [60]. Our data suggest that within growing liver metastases, infiltrating neutrophils progressively adopt an N2 phenotype as the lesions grow and that the greatest concentration of N2 neutrophils resides immediately at the margin of the metastatic lesions.

## Conclusions

Our studies highlight the multifaceted role played by innate immune cells, especially the Ly-6G^+^ component, in regulating the spread of breast cancer cells to specific organs, such as the liver. The role of neutrophils is becoming increasingly important in light of clinical studies that indicate the presence of neutrophil markers or elevated neutrophil to lymphocyte ratios are associated with poor clinical outcomes in breast cancer patients [86,87].

## Additional files

Additional file 1:
**List of 395 differentially expressed genes.** The table contains a list of 395 genes that are differentially expressed between the distinct 4T1-derived metastatic explant populations (bone, lung and liver). Additional file 2:
**Neutrophils are recruited to breast cancer-derived lung and liver metastases.** Paraffin-embedded sections from primary breast tumors, bone, lung and liver metastases were obtained following experimental metastasis assays and subjected to immunohistochemical staining with anti-neutrophil elastase (NE) antibodies. **(A)** Representative images from 20X and 40X magnifications for each metastatic site are shown. 40X images were taken either at the margin of the metastatic lesions (40X margin) or in regions distal to the metastases (40X adj.). **(B)** Positivity of NE staining (expressed as a ratio of positive pixels over the total pixels per field) was quantified within the metastatic lesions (TUMOR), in close proximity of the metastases (PROX ADJ), in tissue adjacent (ADJ) to the metastatic lesions or in control (CTRL) samples lacking breast cancer metastases. Increased recruitment of NE+ cells proximal to liver metastases was routinely observed (^*^: liver tumor vs. liver adj., *P* = 0.002; liver adj. vs. liver prox. Adj., *P* <0.001; lung tumor vs. lung adj., *P* <0.001). Scale bar represents 40 μm (20X) or 20 μm (40X) and applies to all panels of the same magnification. Additional file 3:
**Neutrophils are recruited to early lesions and are maintained at the margin of liver metastases over time.** Paraffin-embedded sections from liver metastases were collected at early (1.5 weeks) or late (3 weeks) time points following splenic injection of breast cancer cells and subjected to immunohistofluorescence staining with anti-S100a8 (red) or anti-neutrophil elastase (green) antibodies. Representative images captured at 63X magnification for each time point are shown. Images were taken either within the metastatic lesions (tumor), at the margin of the metastatic lesions (margin) or in regions distal to the metastases (distal). Scale bar represents 50 μm and applies to all panels. Additional file 4:
**N2-polarized neutrophils are recruited at the invasive front of breast liver metastases.** OCT-embedded sections from liver samples collected 1 week following splenic injection of breast cancer cells were subjected to immunohistofluorescence staining with anti-Ly-6G (cyan), MMP9 (red) or Cd11b (green) antibodies. Representative images captured at 63X magnification for each time point are shown. Images were taken either at the invasive front of the metastatic lesions or in regions distal to the metastases (distal). Arrows: neutrophils (Cd11b^+^/Ly-6G^+^); Arrowheads: pro-tumorigenic (N2)-polarized neutrophils (Cd11b^+^/Ly-6G^+^/MMP9^+^). Dotted lines circumscribe breast cancer metastatic lesions within the liver. Scale bar represents 50 μm and applies to all panels. Additional file 5:
**N2-polarized neutrophils are recruited at the invasive front of breast liver metastases.** OCT-embedded sections from livers with a low metastatic burden were collected at 2 weeks following splenic injection of breast cancer cells and subjected to immunohistofluorescence staining with anti-Ly-6G (cyan), MMP9 (red) or Cd11b (green) antibodies. Representative images captured at 63X magnification for each time point are shown. Images were taken either at the invasive front of the metastatic lesions or in regions distal to the metastases (distal). Arrows: neutrophils (Cd11b^+^/Ly-6G^+^); Arrowheads: pro-tumorigenic (N2)-polarized neutrophils (Cd11b^+^/Ly-6G^+^/MMP9^+^). Dotted lines circumscribe breast cancer metastatic lesions within the liver. Scale bar represents 50 μm and applies to all panels.

## Abbreviations

ADJ: adjacent
BSA: bovine serum albumin
CM: conditioned media
CTRL: control
DAPI: 4′, 6′-diamidino-2-phenylindole
FACS: fluorescence-activated cell sorting
FBS: fetal bovine serum
IgG: immunoglobulin G
iMCs: immature myeloid cells
i.p.: intraperitoneal
IPA: ingenuity pathway analysis
mAb: monoclonal antibody
MDSCs: myeloid derived suppressor cells
N1: anti-tumorigenic phenotype
N2: pro-tumorigenic phenotype
NE: neutrophil elastase
PBS: phosphate-buffered saline
PMN: polymorphonuclear
PROX ADJ: proximal adjacent
qPCR: quantitative PCR
TAMs: tumor-associated macrophages
TANs: tumor-associated neutrophils
μCT: micro-computed. tomography
