# Supplementary material for: Granulocytic immune infiltrates are essential for the efficient formation of breast cancer liver metastases
Source: Breast Cancer Res. 2015 Mar 27;17(1):45. doi: 10.1186/s13058-015-0558-3 (PMC4413545; doi:10.1186/s13058-015-0558-3)
Supplement: Additional file 2: — Neutrophils are recruited to breast cancer-derived lung and liver metastases. Paraffin-embedded sections from primary breast tumors, bone, lung and liver metastases were obtained following experimental metastasis assays and subjected to immunohistochemical staining with anti-neutrophil elastase (NE) antibodies. (A) Representative images from 20X and 40X magnifications for each metastatic site are shown. 40X images were taken either at the margin of the metastatic lesions (40X margin) or in regions distal to the metastases (40X adj.). (B) Positivity of NE staining (expressed as a ratio of positive pixels over the total pixels per field) was quantified within the metastatic lesions (TUMOR), in close proximity of the metastases (PROX ADJ), in tissue adjacent (ADJ) to the metastatic lesions or in control (CTRL) samples lacking breast cancer metastases. Increased recruitment of NE+ cells proximal to liver metastases was routinely observed (*: liver tumor vs. liver adj., P = 0.002; liver adj. vs. liver prox. Adj., P <0.001; lung tumor vs. lung adj., P <0.001). Scale bar represents 40 μm (20X) or 20 μm (40X) and applies to all panels of the same magnification. [file 13058_2015_558_MOESM2_ESM.pptx]

## Slide 1
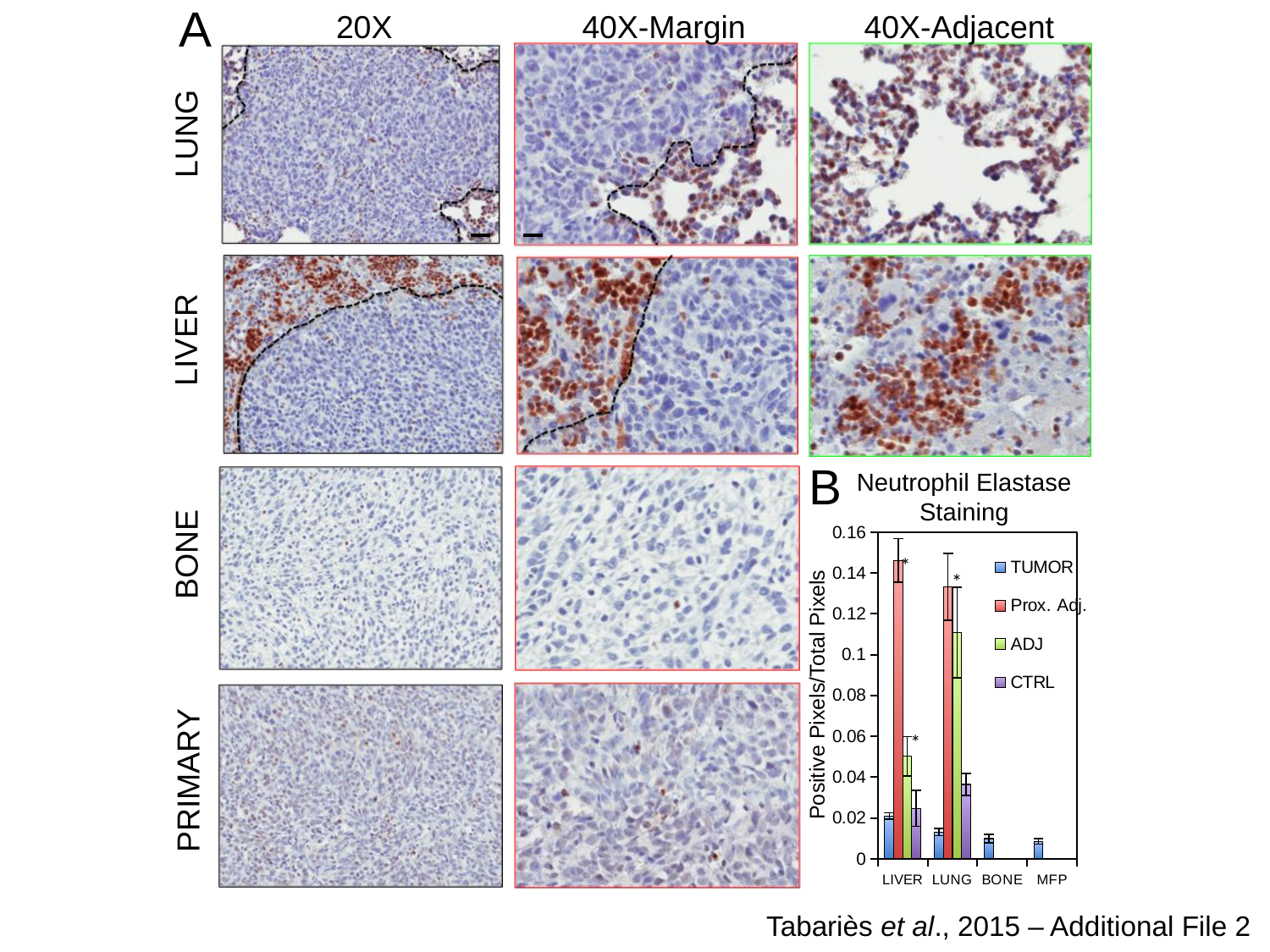

A
20X
40X-Margin
40X-Adjacent
LUNG
LIVER
B
Neutrophil Elastase
Staining
### Chart
| Category | TUMOR | Prox. Adj. | ADJ | CTRL |
|---|---|---|---|---|
| LIVER | 0.0209775235256514 | 0.146246344971316 | 0.0501933036354021 | 0.0246748106615246 |
| LUNG | 0.0130796202015086 | 0.133136566502797 | 0.110858277403514 | 0.0364188505486717 |
| BONE | 0.00986635268855026 | None | None | None |
| MFP | 0.00859895773371792 | None | None | None |BONE
*
*
Positive Pixels/Total Pixels
PRIMARY
*
Tabariès et al., 2015 – Additional File 2
