# Supplementary figures and images for: Granulocytic immune infiltrates are essential for the efficient formation of breast cancer liver metastases
Source: Breast Cancer Res. 2015 Mar 27;17(1):45. doi: 10.1186/s13058-015-0558-3 (PMC4413545; doi:10.1186/s13058-015-0558-3)

## Slide 1
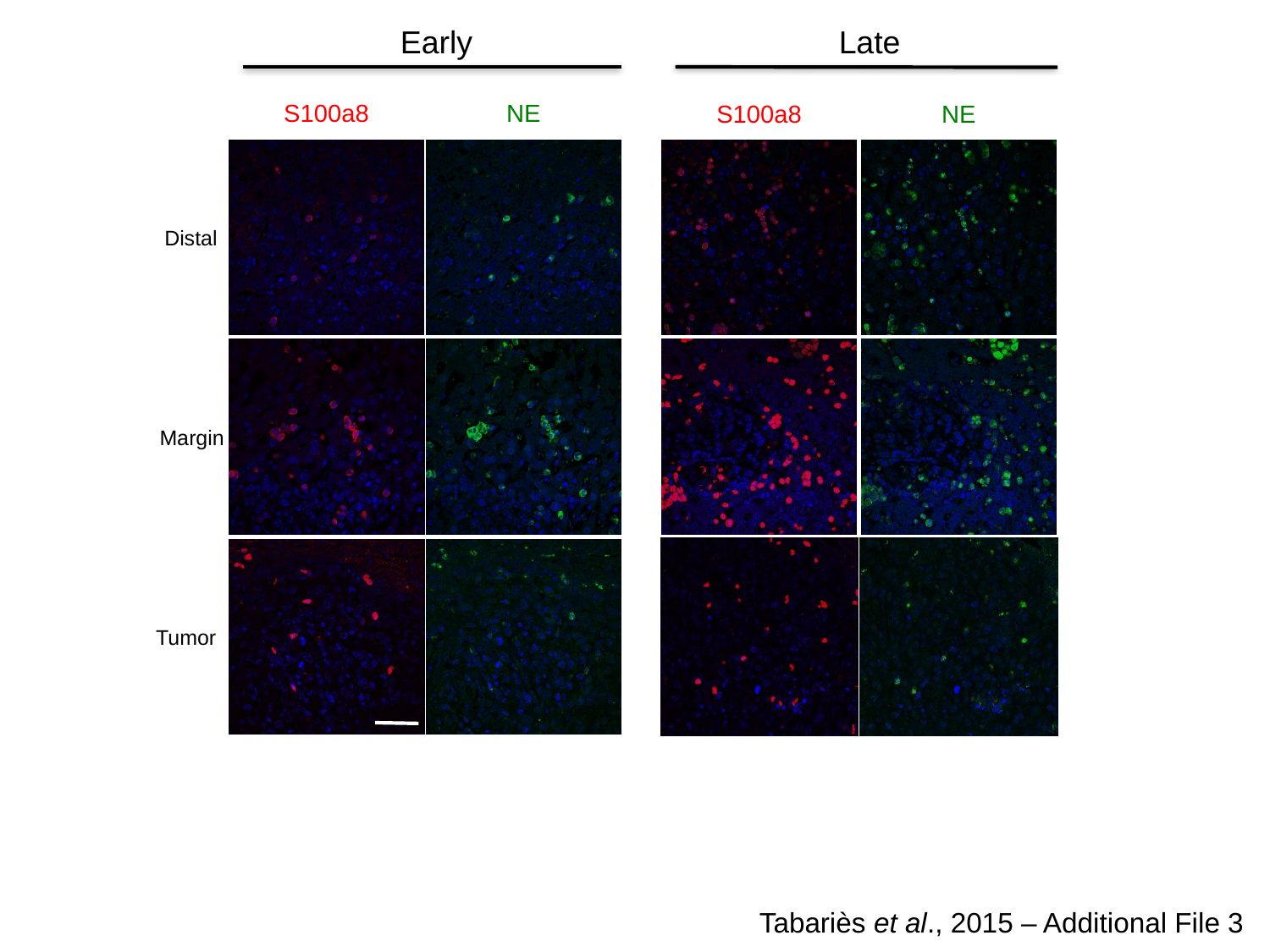

Early
Late
S100a8
NE
S100a8
NE
Distal
Margin
Tumor
Tabariès et al., 2015 – Additional File 3

Supplement: Additional file 3: — Neutrophils are recruited to early lesions and are maintained at the margin of liver metastases over time. Paraffin-embedded sections from liver metastases were collected at early (1.5 weeks) or late (3 weeks) time points following splenic injection of breast cancer cells and subjected to immunohistofluorescence staining with anti-S100a8 (red) or anti-neutrophil elastase (green) antibodies. Representative images captured at 63X magnification for each time point are shown. Images were taken either within the metastatic lesions (tumor), at the margin of the metastatic lesions (margin) or in regions distal to the metastases (distal). Scale bar represents 50 μm and applies to all panels. [file 13058_2015_558_MOESM3_ESM.pptx]
