# Supplementary material for: Granulocytic immune infiltrates are essential for the efficient formation of breast cancer liver metastases
Source: Breast Cancer Res. 2015 Mar 27;17(1):45. doi: 10.1186/s13058-015-0558-3 (PMC4413545; doi:10.1186/s13058-015-0558-3)
Supplement: Additional file 5: — N2-polarized neutrophils are recruited at the invasive front of breast liver metastases. OCT-embedded sections from livers with a low metastatic burden were collected at 2 weeks following splenic injection of breast cancer cells and subjected to immunohistofluorescence staining with anti-Ly-6G (cyan), MMP9 (red) or Cd11b (green) antibodies. Representative images captured at 63X magnification for each time point are shown. Images were taken either at the invasive front of the metastatic lesions or in regions distal to the metastases (distal). Arrows: neutrophils (Cd11b+/Ly-6G+); Arrowheads: pro-tumorigenic (N2)-polarized neutrophils (Cd11b+/Ly-6G+/MMP9+). Dotted lines circumscribe breast cancer metastatic lesions within the liver. Scale bar represents 50 μm and applies to all panels. [file 13058_2015_558_MOESM5_ESM.pptx]

## Slide 1
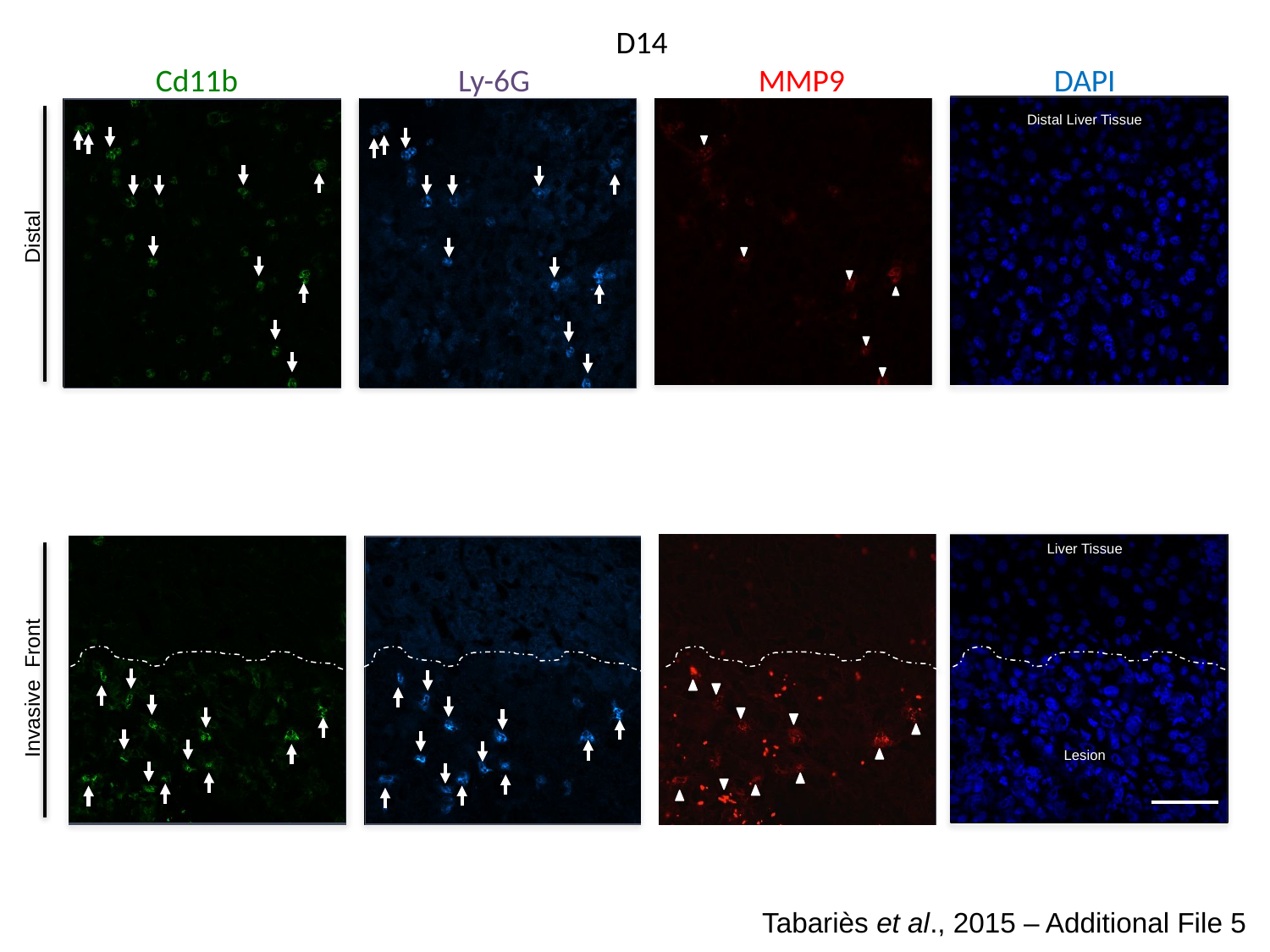

D14
Cd11b
Ly-6G
MMP9
DAPI
Distal Liver Tissue
Distal
Liver Tissue
Lesion
Invasive Front
Tabariès et al., 2015 – Additional File 5
